# Kynurenine, Kynurenic Acid, Quinolinic Acid and Interleukin-6 Levels in the Serum of Patients with Autism Spectrum Disorder

**DOI:** 10.3390/medicina59111906

**Published:** 2023-10-27

**Authors:** Veli Yildirim, Seref Simsek, Ihsan Cetin, Recep Dokuyucu

**Affiliations:** 1 Special Clinic, Department of Child Psychiatry, Yenişehir, Mersin 33110, Turkey; drveliyildirim@gmail.com; 2 Special Clinic, Department of Child Psychiatry, Antalya 07000, Turkey; drserefsimsek@gmail.com; 3 Department of Nutrition and Dietetics, School of Health Sciences, Batman University, Batman 72040, Turkey; cetiniihsan@gmail.com; 4 Department of Physiology, School of Medicine, Atlas University, Istanbul 34413, Turkey

**Keywords:** autism spectrum disorders, kynurenic acid, kynurenine pathway, quinolinic acid, IL-6

## Abstract

*Background and Objectives*: It is known that inflammatory processes play a role in the pathogenesis of autism spectrum disorder (ASD). It is also reported that immune activation induces the kynurenine pathway (KP), as known as the tryptophan destruction pathway. In our study, we aimed to investigate whether the serum levels of KP products and interleukin (IL)-6 activating indolamine 2–3 dioxygenase (IDO) enzyme are different in healthy developing children and children with ASD. *Materials and Methods*: Forty-three ASD children aged 2–9 were included in this study. Forty-two healthy developing children, similar to the patient group in terms of age and gender, were selected as the control group. Serum levels of kynurenic acid, kynurenine, quinolinic acid and IL-6 were analyzed using the ELISA method. ASD severity was evaluated with the Autism Behavior Checklist (ABC). *Results*: The mean age of children with ASD was 42.4 ± 20.5 months, and that of healthy controls was 48.1 ± 15.8 months. While the serum levels of kynurenic acid, kynurenine and interleukin-6 were higher in the group with ASD (*p* < 0.05), there was no significant difference (*p* > 0.05) in terms of the quinolinic acid level. There was no significant difference between the ABC total and subscale scores of children with ASD and biochemical parameters (*p* > 0.05). *Conclusions*: We conclude that these biomarkers must be measured in all ASD cases. They may be important for the diagnosis of ASD.

## 1. Introduction

Autism spectrum disorder (ASD) is defined as a neurodevelopmental disorder with limited, repetitive behavior, interests and activities, as well as inadequate social contact and interaction [1]. Although the etiology of ASD has not been fully elucidated so far, it is thought to occur due to genetic, environmental and immunological factors [2].

In recent years, studies on the immune system in ASD have been increasing. It is reported that there is a link between genes encoding immune system-related proteins (immune-related proteins) and ASD. It is thought that immune system abnormalities in children with ASD may have negative effects on brain development and the development of synaptic functions [3]. However, in cases with ASD, it is stated that the synergistic effect of genetic predisposition and immune dysfunction on neuronal function, migration and proliferation occurs via cytokines [4]. In a meta-analysis, it was reported that compared to healthy controls, children with ASD had significantly higher serum levels of IL-6, interleukin (IL)-1 beta, interferon-gamma, IL-8, eotaxin, and monocyte chemotactic protein-1. However, they had significantly lower TGF-β levels [5]. Other studies have shown that IL-6 is one of the most markedly increased cytokines in ASD [4,5,6]. IL-6 induces indolamine 2–3 dioxygenase (IDO) enzyme, which plays a key role in the kynurenine pathway (KP) [7,8].

KP metabolites and inflammation markers have been studied in attention deficit hyperactivity disorder (ADHD), depressive disorder, bipolar disorder, schizophrenia, and neurodegenerative diseases [9,10,11]. In a study conducted in adult schizophrenia patients, the increase in the level of IL-6 with kynurenine and kynurenic acid showed that the immune system had an effect on this pathway [12]. Considering the place of immunological processes in the etiology of ASD, Lim et al. presented the only study to date where KP and cytokines were studied simultaneously [13]. Lim et al. found that the tryptophan level did not change in children with ASD, while the kynurenine level, quinolinic acid level and kynurenine/tryptophan ratio increased and the picolinic acid level decreased compared to the siblings of children with ASD [13]. Gevi et al. [14] reported that the level of plasma kynurenine was unchanged in children with ASD compared to healthy controls.

Kynurenic acid formed during KP antagonizes the N-methyl-D-aspartate (NMDA) receptor at low concentrations and shows a neuroprotective effect by competing with the coagonist of this receptor, glycine [15,16]. On the other hand, quinolinic acid formed during KP creates a neurotoxic effect through NMDA receptor agonism [16,17]. In addition, kynurenic acid antagonizes cholinergic alpha-7 nicotinergic receptors in a non-competitive way [15,16]. However, kynurenic acid has been shown to affect glutamate, dopamine, and γ-aminobutyric acid (GABA) extracellular levels bi-directionally [13,18,19].

In our study, we aimed to analyze whether there is a difference between children with ASD and healthy developing children in terms of kynurenic acid, kynurenine, quinolinic acid and IL-6, which activates IDO enzyme. In addition, we aimed to investigate whether there are differences in terms of biochemical parameters between children with regressive-type and non-regressive-type ASD.

## 2. Materials and Methods

### 2.1. Ethics Statement and Experimental Design

This research was approved by the Dicle University Local Clinical Research Ethics Committee. Our study was carried out in accordance with the Declaration of Helsinki. This study was carried out between May 2015 and October 2015 at the Department of Child and Adolescent Mental Health and Diseases, Dicle University Hospital. Participants were informed about the study. Informed consent was obtained from the parents of the children in order for them to participate in this voluntary study. The study group included 43 children aged 2–9 diagnosed with ASD according to DSM-5 criteria.

Cases accepted as ASD in the study were recorded consecutively and the results of the interviews were noted at least twice every six months by two specialist psychiatrists. Forty-two children of similar age and gender, living at similar addresses to the patients, without a history of psychiatric disease and developmental delay, were selected as the healthy control group. For the control group, intellectual disability, ASD, certain language impairment, the presence of a neurological disease, and clinically active infection were selected as exclusion criteria. Hearing tests were applied to all participants. Patients with dysmorphic appearance, medical disorders including Fragile X, Rett syndrome, epilepsy, and clinically active infections were excluded. Psychostimulants were used in one patient due to the additional diagnosis of ADHD, and antipsychotic drugs were used in another case due to the additional diagnosis of a behavioral disorder. There was a similar history of vaccination in both patient and control groups.

### 2.2. Autism Behavior Checklist (ABC)

ABC was developed by Krug et al. [20] in 1993. It has been used to assess the severity of autism symptoms. It consists of five 57-item subscales: ABC language skills, relationship building, sensory, body and object use, and self and social care skills. The lowest score of the scale is 0, and the highest score is 159.

### 2.3. Biochemical Measurements

The serum levels of kynurenic acid, kynurenine and quinolinic acid were determined with the ELISA method (SUNRED Biotechnology Co., Ltd., Shanghai, China), and the serum levels of interleukin-6 was determined with the ELISA method (Shanghai LZ Biotech Co., Ltd., Shanghai, China) according to the manufacturer’s instructions.

### 2.4. Statistical Analysis

SPSS (Statistical Package for Social Sciences, IBM Corp, Armonk, NY, USA) 18.0 package program was used all statistical analysis. The Shapiro–Wilk test was used to assess the data normality. The arithmetic mean values and median values between two groups were compared using the Mann–Whitney U and Student *t* tests, and *p* values < 0.05 were considered as statistically significant. A chi-square test was used for categorical data comparisons. The significance level was chosen as *p* < 0.05.

## 3. Results

In our study, the mean age of the patient group was 42.4 ± 20.5 months, and the mean age of the control group was 48.1 ± 15.8 months (Table 1). There was no statistically significant difference between the groups in terms of age, gender, parental age, number of siblings, sibling rank, and kinship ratio between parents (*p* > 0.05). On the other hand, the incidence of psychiatric disease in the family and close relatives was significantly higher in the patient group than in the mothers of the controls (*p* = 0.01).

The biochemical parameters of the autistic and non-autistic cases are shown in Table 2. Kynurenic acid, kynurenine, kynurenic acid/quinolinic acid ratio and interleukin-6 levels were significantly higher in the patient group compared to the controls (*p* = 0.017, *p* = 0.006, *p* = 0.002 and *p* = 0.001, respectively).

The quinolinic acid levels were lower in the patient group compared to the controls, but not statistically different (*p* = 0.118) (Table 3).

There was no significant difference between patients with and without regression in terms of kynurenic acid, kynurenine, quinolinic acid and interleukin-6 levels (*p* = 0.28, *p* = 0.56, *p* = 0.27, *p* = 0.36, respectively).

ABC scores were measured in the patient group and were as follows: sensory, 9.1 ± 3.5; relating (social skills), 20.0 ± 6.0; body and object use, 17.3 ± 6.2; language, 18.4 ± 6.0; social and self-help, 12.5 ± 3.7, with a total of 77.3 ± 22.1.

A regressive autism type was observed in 21% (*n* = 9) of ASD cases. Socio-demographic characteristics of patients with and without regression were similar in the patient group (*p* > 0.05). There was no significant difference between patients with and without regression in terms of the ABC total and subscales scores (*p* > 0.05).

The relationship between biochemical parameters and ABC scores was also assessed. There was no significant relationship between biochemical parameters and ABC total and subscale scores (*p* > 0.05).

## 4. Discussion

In our study, kynurenic acid, kynurenine, kynurenic acid/quinolinic acid and IL-6 levels were significantly increased in children with ASD compared to the control group. However, the regressive autism type was observed in 21% (*n* = 9) of ASD cases. The socio-demographic characteristics of patients with and without regression were similar in the patient group. There was no significant difference between patients with and without regression in terms of the ABC total and subscales scores (*p* > 0.05). The products formed in KP have been reported to be closely related to neurotransmitters such as dopamine, serotonin and glutamate [13,17,18,19]. These neurotransmitters are known to play a role in the etiopathogenesis of ASD [21]. Tryptophan, the substrate of KP, is very important for ASD, because abnormalities of tryptophan in metabolic pathways other than KP may play a role in the etiology of ASD [12,21]. Serotonin formed as a result of tryptophan metabolism is the first biomarker found in children with ASD [22]. Moreover, serotonergic and dopaminergic systems have been shown to affect ASD and modulate each other [23]. Dopamine and its receptors formed with phenylalanine and tyrosine metabolism have been found to display some changes in children with ASD [24,25]. In addition, it has been stated that the imbalance between excitatory glutamate and inhibitor GABA in ASD develops in the direction of excitoxicity or hyperexication [26,27]. The reporting of excitatory glutamate at the highest levels in the second year of life of children with ASD supported its importance in early development neurologically [26,27].

Lim et al. also emphasized the relationship between KP and glutamate [13]. It has been reported that at an age of three years, when autistic regression is most common, this relationship may be related to immune processes and glutamate may be related to antibodies against the NMDA receptor [28]. In addition, IL-6, which activates KP, has been shown to increase with NMDA receptor antibodies [29]. The relationship between early ages, immunological processes and KP seems to be very important for the glutamatergic system. Considering the emphasis on the relationship between glutamate and KP relationship provided by Lim et al. [13], a study on kynurenic acid, IL-6 and NMDA receptor antibodies, which are related to NMDA receptors, may be interesting. In the current study, there is not significant difference between the levels of KP metabolites in children with autistic regression and those with a glutamatergic system, which suggests that further studies should be performed on this subject. Studying inflammation markers, KP metabolites, and antibodies against the NMDA receptor in a larger group with autistic regression may yield meaningful findings on this subject. Considering autoimmune infections in children with autism, it may be useful to study autoimmune thyroiditis, which is common, and KP metabolites.

As with glutamatergic development, the relationships between serotonergic development and tryptophan metabolism and KP have been demonstrated [30]. Serotonin peaks in the brain in the second month before birth and shows a slight decrease up to three years after birth [30]. The 5-hydroxy indole acetic acid level in the cerebrospinal fluid with serotonin metabolites was found to be higher in children than in adults [30]. It has been stated that serotonin may pass from the mother’s blood to the baby during the intrauterine period, but there may be neuropathological developments in babies with ASD in the first few months of life, in which KP can work more actively in tryptophan metabolism [21]. It can be concluded that the relationship of both serotonergic and glutamatergic development with KP at an early age may be important for ASD.

Lim et al. [13] compared glutamatergic activity, IL-6 and KP products with 15 ASD children and their 12 healthy siblings. Lim et al. stated that the picolinic acid level, with neuroprotective effects, is low in the serum, and a high quinolinic acid level, with neurotoxic effects, may be associated with abnormal development of the brain in ASD. However, Sweeten et al. [31] did not find the level of kynurenine to be at different levels in children with ASD than in healthy controls. In our study, kynurenine and kynurenic acid levels were higher in the patient group. Although it is known that kynurenic acid has neuroprotective effects in physiological conditions, the increase in the kynurenic acid level here may have neurotoxic effects [29]. It is mentioned that the glutamatergic system and neuroinflammation processes start at early ages and even in the womb [31]. In addition, Lim et al. [13] determined that the kynurenic acid level was not different from the healthy control group, and that the quinolinic acid levels were high. Many studies performed on schizophrenia patients with neuroinflammation have shown elevated kynurenic acid levels [8,15,32,33]. It has also been reported in recent years that kynurenic acid may play a role in inflammatory neurological and behavioral disorders [16]. Many studies have shown that high levels of kynurenic acid exist in patients with impaired cognitive function [34,35,36]. Cognitive disorders, which may be associated with the glutamatergic system, are very common problems in ASD [37]. Considering cognitive disorders and neuroinflammation in ASD, it is thought that the serum level of kynurenic acid is important in younger ages based on the data in our study. Immune system function assessments other than IL-6, another inflammation marker, blood count, allergic diseases, gestational infection, gestational medicine uses, gestational weight/age, breast/formula feeding and if the mothers experienced gestational or post-partum depression must be evaluated in future research investigations.

In our study, it was found that this rate increased significantly in children with ASD. A high presence of kynurenic acid and kynurenine suggests that the IDO enzyme is activated. IDO enzyme activity has been shown to be induced when IL-6 is administered to the hippocampus of rats [8]. It has also been reported that when cortical astrocyte cells are administered IL-6, the kynurenic acid level increases, and there is a close interaction between IL-6 and KP [38,39]. The increased IL-6 and kynurenic acid levels in our study suggest that there is an abnormal immune response in the brain in ASD and aberrant neurotransmission may occur. In addition, increased kynurenic acid levels may be associated with a change in dopaminergic tonus, which plays an important role in the etiopathogenesis of ASD, because kynurenic acid has been reported to correlate negatively with dopaminergic tonus and affect glutamatergic receptors [8]. High kynurenic acid levels have also been shown to impair GABAergic neurotransmission in pyramidal neurons in the hippocampus [40]. In light of this information, we can say that KP can mediate the understanding of the etiopathogenesis of ASD, the evaluation of biomarker candidates and the thinking regarding new treatment approaches.

IL-6 levels and 5HTP serum levels were found to be positively correlated with Childhood Autism rating scale test severity scores [41]. IL17a correlated with autism severity, but no review of other kynurenine pathway products could be found in the literature [42]. In addition, there is a positive correlation between IL-6 and 5-HTP, as well as a correlation between IL-6 and 5HTP [41]. It has been reported that non-steroidal anti-inflammatory (NSAID) and serotonin reuptake inhibitor (SSRI) drugs, which are frequently used in children, affect these cytokines [41]. In studies using 5HTP degradation products, KP or other cytokines, it should be considered that there may be children using NSAIDs or SSRIs. This may be the reason why we could not find a correlation with the severity of ASD in our study. Savitz et al. reported that ibuprofen was neuroprotective and decreased the kynurenic acid (KynA) level, and they measured this effect on the best KynA/QA ratio [12]. A study examining the use of ibuprofen in children with and without ASD should be undertaken, and the rates of autism severity and KynA/QA in children may also be useful in this respect. Therefore, there may not have been a significant difference in both the ABC scores and KynA/QA ratios. Bilgic et al. found that correlations between serum KP metabolites and ABC scores were evaluated in the ASD group, while associations were not [43].

Bryn et al. found that the QA level of children with ASD was 535.77 ± 207.50 nM, while that in controls, 615.17 ± 329.27 nM, was statistically insignificantly different [44]. The level of quinolinic acid (ng/mL) was 13.7 ± 7.4 ng/mL in patients with autism, while the level in healthy controls was 17.7 ± 9.8 ng/mL, consistent with the literature. However, the fact that this difference is not statistically significant may be related to the small number of cases. Bryn and friends found that the QA/KA ratio did not differ between patients and controls, and lower KA may be influential in ASD. In our study, the KA levels were also higher in the control subjects. Bilgiç et al. observed that high levels of KA provide neuroprotective and anxiolitic effects [43]. It can be concluded that it would be more useful to look at each metabolite in a proportional way to show the enzyme activities rather than individually.

It was determined that while the serum level of KynA decreased and that of QA increased in rats who were socially isolated for eight weeks, this situation was reversed with clozapine, an antipsychotic drug [45]. In this respect, it is important that patients with ASD and the controls receive education and do not encounter situations that may cause social isolation, such as screen exposure and parental depression. In our study, it could be considered whether the cases benefited from education and therapies for ASD or whether these therapies were important for their quality of life, and this might have had an effect on KynA/QA ratios. Evaluating social isolation in first-diagnosed ASD cases and comparing the levels of HF products with the levels obtained after training and therapy represents another important study area.

In our study, there are some limitations. First, the sample size was small. Second, inflammatory cytokines other than IL-6 and toxic intermediates in KP other than quinolinic acid were not evaluated. Third, the intelligence and developmental levels of the children with autism included in our study were not evaluated. The absence of tryptophan levels limited the evaluation of KP activation. Third, the fact that the control group was not made up of siblings of autistic children created difficulty in distinguishing the difference that genetic factors may cause. Fourth, those being administered NSAIDs, SSRIs, psychostimulants, antipsychotics, other psychotropic drugs, and vaccines and with inflammatory diseases were included in the study. In contrast, the strengths of our study are that the mean age of the patients was lower and the range was narrower than the sample group in studies on KP in ASD until now. Moreover, the fact that IL-6 is studied in this paper and the evaluation of the inflammatory processes and their relationship with KP is performed can be considered as a superior aspect.

## 5. Conclusions

In conclusion, the serum levels of kynurenic acid, kynurenine and interleukin-6 were higher in children with ASD than in the control group. Therefore, these biomarkers must be measured in all ASD cases. They may be important for the diagnosis of ASD. We could not find any difference among the cases because of the small number of regressive-type cases (nine) and the lack of change in kynurenine pathway products. This may be related to the low number of cases with autism displayinh a regressive phase. It can be suggested that many other neuroinflammation markers and KP metabolites must be studied simultaneously in the future. In addition, further studies with more extensive sampling are required.

## Figures and Tables

**Table 1:** Data related to some sociodemographic variables in subjects with and without autism.

	ASD (*n* = 43)	Control (*n* = 42)	*t* Value	*p* Value
Age (months)	42.4 ± 20.5	48.1 ± 15.8	−1.423	0.16
Sex (M/F)	37/6	33/9		0.54
Mother’s age (years)	30.8 ± 5.7	32.5 ± 5.1	−1.418	0.16
Father’s age (years)	35.5 ± 5.6	36.8 ± 5.1	−1.141	0.26
Number of siblings	3.0 ± 1.2	3.3 ± 1.4	−1.024	0.31
Order of birth	2.7 ± 1.3	2.9 ± 1.4	−0.691	0.49
History of psychiatric disorders * (yes/no)	10/33	1/41		**0.01**
Consanguineous marriage (yes/no)	14/29	12/30		0.87

* History of psychiatric disorders in the immediate family and first-degree relatives.

**Table 2:** Biochemical parameters in subjects with and without ASD.

	ASD (*n* = 43)	Control (*n* = 42)	*t* Value	*p* Value
Kynurenic acid (nmol/mL)	4.6 ± 1.6	3.8 ± 1.9	2.178	**0.017**
Kynurenine (nmol/mL)	1986.7 ± 894.9	1492.1 ± 914.3	2.520	**0.006**
Quinolinic acid (ng/mL)	13.7 ± 7.4	17.7 ± 9.8	−2.136	0.118
Kynurenic acid/Quinolinic acid	0.45 ± 0.22	0.29 ± 0.19	3.143	**0.002**
Interleukin-6 (ng/L)	260.3 ± 72.1	193.2 ± 90.9	3.775	**0.001**

**Table 3:** Patients with and without regression in terms of kynurenic acid, kynurenine, quinolinic acid and interleukin-6.

	ASD with Regression (*n* = 9)	ASD without Regression (*n* = 34)	*p* Value
Kynurenic acid (nmol/mL)	5.1 ± 1.3	4.7 ± 2.1	0.28
Kynurenine (nmol/mL)	2090.1 ± 941.6	1825.8 ± 519.3	0.56
Quinolinic acid (ng/mL)	15.7 ± 6.9	13.2 ± 8.7	0.27
Interleukin-6 (ng/L)	272.2 ± 82.1	257.1 ± 60.6	0.36

## Data Availability

The data that support the findings of this study are available from the corresponding author upon reasonable request.
